# Echovirus induces autophagy to promote viral replication *via* regulating mTOR/ULK1 signaling pathway

**DOI:** 10.3389/fimmu.2023.1162208

**Published:** 2023-04-11

**Authors:** Chunchen Wu, Luzhi Zeng, Wenfu Yi, Yuanjiu Miao, Yihan Liu, Qiming Wang, Shi Liu, Guoping Peng, Zhenhua Zheng, Jianbo Xia

**Affiliations:** ^1^ Department of Laboratory Medicine, Maternal and Child Health Hospital of Hubei Province, Tongji Medical College, Huazhong University of Science and Technology, Wuhan, China; ^2^ College of Bioscience and Biotechnology, Hunan Agricultural University, Changsha, China; ^3^ CAS Key Laboratory of Special Pathogens and Biosafety, Center for Emerging Infectious Diseases, Wuhan Institute of Virology, Chinese Academy of Sciences, Wuhan, China; ^4^ State Key Laboratory of Virology, Modern Virology Research Center, College of Life Sciences, Wuhan University, Wuhan, China

**Keywords:** enterovirus, echovirus, autophagy, viral replication, virus-host interaction

## Abstract

Among enteroviruses, echovirus can cause severe illnesses in neonates or infants, with high morbidity and mortality. Autophagy, a central component of host defense mechanisms, can function against diverse infections. In the present study, we investigated the interplay between echovirus and autophagy. We demonstrated that echovirus infection increases LC3-II expression dose-dependently, accompanied by an increased intracellular LC3 puncta level. In addition, echovirus infection induces the formation of autophagosome. These results suggest that echovirus infection induces autophagy machinery. Furthermore, phosphorylated mTOR and ULK1 were both decreased upon echovirus infection. In contrast, both levels of the vacuolar protein sorting 34 (VPS34) and Beclin-1, the downstream molecules which play essential roles in promoting the formation of autophagic vesicles, increased upon virus infection. These results imply that the signaling pathways involved in autophagosome formation were activated by echovirus infection. Moreover, induction of autophagy promotes echovirus replication and viral protein VP1 expression, while inhibition of autophagy impairs VP1 expression. Our findings suggest that autophagy can be induced by echovirus infection *via* regulating mTOR/ULK1 signaling pathway and exhibits a proviral function, revealing the potential role of autophagy in echovirus infection.

## Introduction

Human enteroviruses (HEVs) are small, nonenveloped, positive single-strand RNA viruses belonging to the genus *Enterovirus* within the family *Picornaviridae*. More than 100 enterovirus types have been identified and classified into four species, A to D, according to molecular and antigenic properties (1). Echovirus was discovered when the first tissue-culture techniques were introduced into laboratories (2). Currently, echovirus has been classified within the B species, the largest group of the *Enterovirus* genus, together with coxsackievirus group B, coxsackie A9, and several novel enteroviruses. Like other enteroviruses, echovirus infections are associated with a broad spectrum of illnesses, ranging from minor symptoms (e.g., febrile rash, mild hand, foot, and mouth diseases (HFMD)) to severe, potentially fatal conditions (e.g., aseptic meningitis, encephalitis and acute flaccid paralysis (AFP)) (3). Among 30 serotypes, echovirus 11 was one of the most commonly identified serotypes that cause severe illnesses in neonates or infants, with high morbidity and mortality (4–8). In addition, echovirus 11 has frequently been found to be associated with outbreaks in neonatal intensive units (NICUs) or postpartum care centers, causing public health threats globally (9–12). However, the pathogenic mechanisms of echovirus are poorly understood, limiting the development of antiviral strategies against echovirus.

Autophagy is an evolutionarily conserved intracellular degradation process by which misfolded proteins, damaged organelles, and various invading pathogens are sequestered in the cytoplasm and removed to maintain cellular homeostasis (13). A key initial event in autophagy is the formation of the autophagosome, a unique double-membrane organelle that engulfs the cytosolic cargo destined for degradation. A series of autophagy-related genes (ATG) has been identified to participate in these processes (14). As a part of autonomous innate immunity, autophagy functions to defend individual cells against invading pathogens such as bacteria, fungi, parasites, and viruses (15, 16). For example, autophagy can exert antiviral functions during Sindbis virus or tobacco mosaic virus infection by selectively targeting viral particles or components to the lysosomes for degradatio (17). However, many viruses, for example, poliovirus, coxsackievirus, and hepatitis C virus, have evolved various strategies to hijack and subvert host autophagy for their life cycles and pathogenesis (18–20). Although previous studies suggested a potential role for autophagy in echovirus 7 entry (21), the interplay between echovirus and autophagy remains unclear.

In the present study, we explored the induction of autophagy machinery during EchoV infection by monitoring the activation of LC3 and the presence of autophagosome-like structures. Also, we checked the effects of inducing or perturbing the autophagy pathway, using pharmacological inducers or inhibitors, respectively, on viral replication. Our data revealed that autophagy is induced during echovirus infection and is involved in echovirus replication.

## Materials and methods

### Cell culture and virus infection

Human rhabdomyosarcoma (RD; CCL-136; ATCC) cells were maintained in Dulbecco’s modified Eagle Medium (DMEM) containing 10% fetal bovine serum (FBS; Life Technologies) at 37°C in a 5% CO2 incubator. Echovirus 11 strain (NCBI Accession No. OP764694) was isolated from a feces sample of a 24-day-old female neonate with enterovirus infection after passaging in the RD cells. RD cells were infected with echovirus at various multiplicity of infection (MOI) for 1.5 h in serum-free DMEM. The cells were washed with phosphate-buffered saline (PBS) and cultured in a completely fresh medium for various times as indicated until they were harvested. For autophagy induction experiments, cells were infected or mock-infected with echovirus for 1.5 h, then cultured in a complete medium containing rapamycin (Selleck, AY-22989) at indicated concentrations for the indicated times. For autophagy inhibition experiments, cells were cultured in DMEM containing indicated concentrations of 3-methyladenine (3-MA) (Selleck, S2767) for 2 h, followed by echovirus infection for 1.5 h, and then incubated with fresh DMEM for 16 h. Cell counting kit-8 (CCK8) (Vazyme, A311-01) assay was performed to examine the cytotoxicity of rapamycin or 3-MA to RD cells.

### Western blotting

Western blotting was performed as described previously (22). The used antibodies were as follows: anti-LC3 (Cell Signaling Technology, 3868), anti-p62 (Proteintech, 18420-1-AP), anti-mTOR (Cell Signaling Technology, 2972), anti-phospho-mTOR (Cell Signaling Technology, 2971), anti-ULK1 (Cell Signaling Technology, 8054), anti-phospho-ULK1 (Ser757) (Cell Signaling Technology, 6888), anti-VPS34 (Proteintech, 12452-1-AP), anti-Beclin-1 (Proteintech, 11306-1-AP) and anti-β-actin (Santa Cruz Biotechnology, sc-47778). Anti-VP1, a rabbit polyclonal antibody, was produced by Wuhan Abclonal Biotechnology. The relative band intensities of the proteins were quantified using the NIH ImageJ software.

### Immunofluorescence microscopy

RD cells were transiently transfected with a GFP-tagged LC3 expression vector (GFP-LC3) as described previously (23) using Lipofectamine (Invitrogen). At 24 h post-transfection, cells were infected with echovirus for 1.5 h, then cultured in a complete medium in the presence or absence of rapamycin for 16 h. The fluorescence of GFP-LC3 was observed under a Nikon A1 confocal fluorescence microscope. The nuclei were stained with Hoechst 33258.

### Transmission electron microscopy

Echovirus-infected cells were fixed with 0.5% glutaraldehyde at 4°C for 10 min and then fixed with 2.5% glutaraldehyde at 4°C for 1 h. After that, cells were further fixed with 0.1% osmium tetroxide. The cells were then dehydrated in a gradient series of ethanol and embedded with LR White (Agar Shin sections were cut on a Leica EM FC7 UC7 ultramicrotome and viewed under an FEI Tecani G20 TWIN transmission electron microscope.

### Real-time PCR

Total RNA was extracted from cells using TRIzol (Invitrogen) and then reverse transcribed into cDNA using FastKing gDNA Dispelling RT SuperMix (TIANGEN, KR118-02). Quantitative reverse transcription-polymerase chain reaction (qRT-PCR) was performed to detect viral RNA using Taq Pro Universal SYBR qPCR Master Mix (Vazyme, Q712-02). The following primers were used: EchoV-F: 5’-AAAGTGG CCAAAGGCAAGTC-3’; EchoV-R: 5’-GGTCAGGATCACACCCAACC-3’; GAPDH-F: 5’-TGGTATCGTGGAAGGACTCA-3’; GAPDH-R: 5’-CCAGTAGAGG CAGGGATGAT-3’. The relative levels of EV-D68 RNA in different samples were determined using a comparative 2^-ΔΔCT^ method and normalized to the GAPDH gene.

### Statistical analyses

Statistical analysis was conducted using GraphPad Prism 5.0 software (GraphPad Software). A one-way ANOVA was used to determine statistically significant differences in multiple comparisons. *P*<0.05 was considered statistically significant. The results are presented as the mean ± standard deviations (n≥3).

## Results

### Echovirus infection induces autophagy

**Figure 1:**
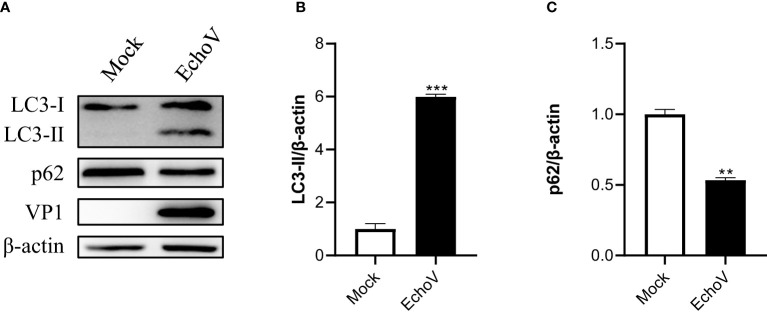
Echovirus infection induces autophagy. RD cells were infected or not (Mock) with echovirus 11 at 1 MOI for 1.5 h. At 16 h post-infection, cells were then harvested. **(A)** Intracellular LC3 and p62 proteins were detected *via* western blotting. β-actin served as a loading control. This result is representative of three independent experiments. The relative band intensities of detected proteins were calculated using ImageJ from NIH, and the result was represented as the ratio of LC3-II to β-actin **(B)** or p62 to β-actin **(C)**. A one-way ANOVA was used to determine statistically significant differences in multiple comparisons. **p<0.01, ***P<0.001.

A hallmark of autophagy induction is that the LC3-I protein undergoes a lipidation modification, and phosphatidylethanolamine (PE) is conjugated to LC3-I to generate LC3-II, which becomes membrane-associated and participates in autophagosome formation (24). To determine whether echovirus infection regulates autophagy, we examined the conversion of endogenous LC3-I to LC3-II. Echovirus-infected cells exhibited an increase in LC3-II and viral VP1 protein when compared with uninfected Mock cells (**Figure 1A**). The densitometry ratio of LC3-II to β-actin showed an increase (**Figure 1B**). In contrast, the autophagic receptor p62 level correspondingly decreased (**Figures 1A, C**). These results suggested that echovirus infection may induce autophagy.

### Echovirus infection induced-autophagy is positively correlated with viral load

**Figure 2:**
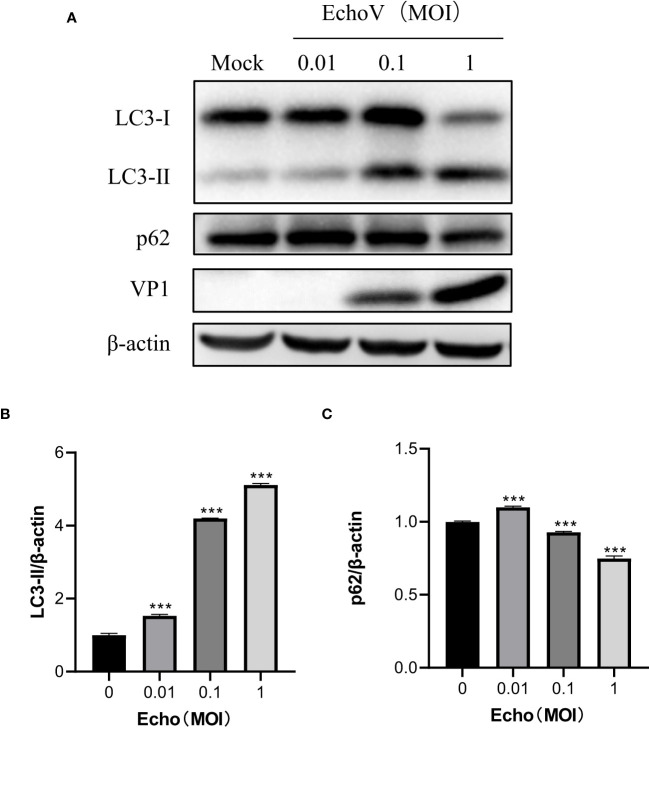
Echovirus infection induced-autophagy is positively correlated with viral load. RD cells were infected or not (Mock) with echovirus 11 at different MOI for 1.5 h. At 16 h post-infection, cells were then harvested. **(A)** Intracellular LC3 and p62 proteins were detected *via* western blotting. β-actin served as a loading control. This result is representative of three independent experiments. The relative band intensities of detected proteins were calculated using ImageJ from NIH, and the result was represented as the ratio of LC3-II to β-actin **(B)** or p62 to β-actin **(C)**. A one-way ANOVA was used to determine statistically significant differences in multiple comparisons. ***P<0.001.

To explore the relationship between viral concentration and the degree of autophagy, RD cells were infected with echovirus at indicated MOIs, and the expression of LC3-II was determined by Western blotting. As the viral MOIs increased, the level of viral capsid protein (VP1) gradually increased. Meanwhile, the level of LC3-II expression gradually increased in echovirus-infected cells. In contrast, the level of p62 expression decreased (**Figure 2A**). The densitometry ratio of LC3-II to LC3-I showed an increase. In contrast, the densitometry ratio of p62 showed a decrease, especially under virus infection at 1 MOI (**Figures 2B, C**). These results indicated that echovirus infection induced-autophagy was positively correlated with viral load.

### Echovirus infection increases the level of LC3 puncta formation

**Figure 3:**
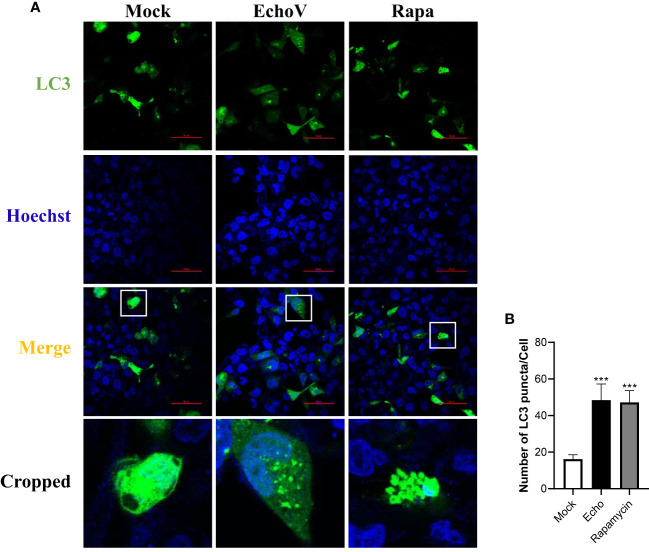
Echovirus infection increases the level of LC3 puncta formation. RD cells were transfected with GFP-LC3 plasmid for 24 h, followed by infected or not (Mock) with echovirus 11 at 1 MOI for 1.5 h. **(A)** At 16 h post-infection, cells were fixed and analyzed by confocal fluorescence microscopy. RD cells were treated with rapamycin (500 nM) (Rapa) for 16 h as a positive control. **(B)** Quantitation of the numbers of LC3 puncta in RD cells. Data shown represent the number of LC3 puncta per cell under each condition. A one-way ANOVA was used to determine statistically significant differences in multiple comparisons. ***P<0.001.

The redistribution of LC3 from a diffuse cytoplasmic localization to a characteristic punctate cytoplasmic pattern, which reflects the recruitment of LC3 to autophagic vesicles, is another hallmark of autophagy (25). Therefore, a GFP-tagged LC3 expression vector (GFP-LC3), as described previously (23), was used to assess other autophagy induced by echovirus infection. In cells transfected with GFP-LC3, the level of LC3 puncta formation was increased by echovirus infection (**Figures 3A, B**). These findings further confirmed that autophagy was induced by echovirus infection.

### Echovirus infection induces autophagosome formation

**Figure 4:**
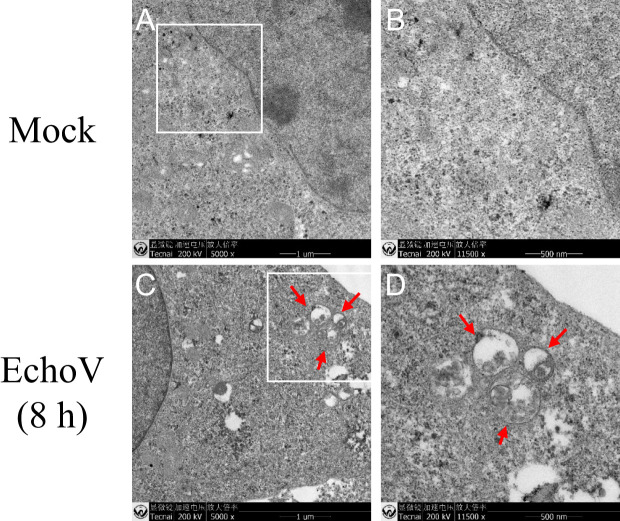
Echovirus infection induces autophagosome formation. RD cells were infected or not (Mock) with echovirus 11 at 1 MOI for 1.5 h. At 8 h post-infection, cells were then fixed and subjected to transmission electron microscopy analysis. Lower magnification of Mock **(A)** and EchoV-infected **(C)** cells. **(B)** Higher magnification of the area in white square of **(A)**; **(D)** Higher magnification of the area in white square of **(C)**. Scale bar in A and C, 1 µm; Scale bar in B and D, 500 nm. The autophagosomes are denoted by red arrows.

A key initial event in autophagy is the formation of the autophagosome, a unique double-membrane organelle that engulfs the cytosolic cargo destined for degradation. Therefore, ultrastructural analysis was performed with RD cells with or without echovirus infection. Double-membrane vesicles engulfing cytosolic materials were observed in the cytoplasm of infected cells but not in uninfected cells under transmission electron microscopy (**Figure 4**). The data revealed that echovirus infection induced autophagosome formation.

### Echovirus infection induces the activation of signaling pathways involved in autophagosome formation

**Figure 5:**
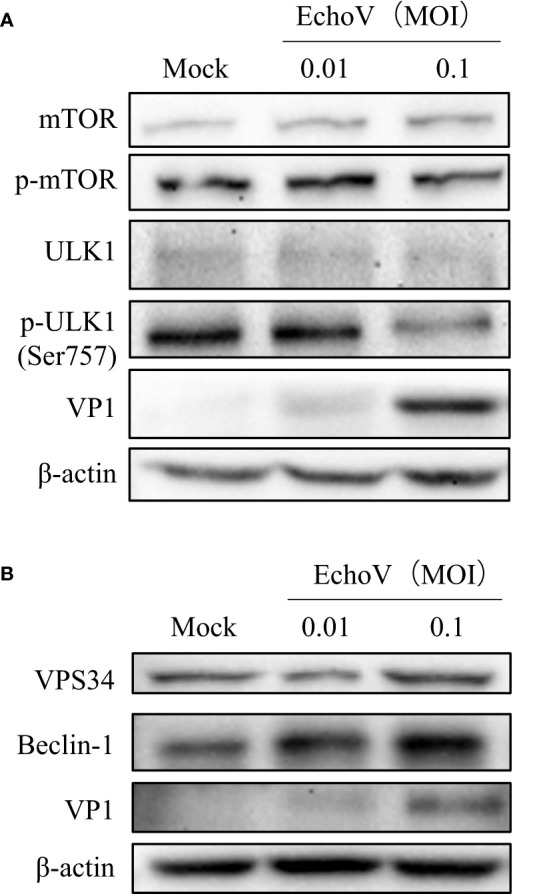
Echovirus infection induces the activation of signaling pathways involved in autophagosome formation. RD cells were infected or not (Mock) with echovirus 11 at different MOI for 1.5 h. At 16 h post-infection, cells were then harvested. **(A)** Intracellular mTOR, phosphorylated mTOR (p-mTOR), ULK1, phosphorylated ULK1 (p-ULK1), and viral VP1 proteins were detected *via* western blotting. **(B)** Intracellular VPS34, Beclin-1, and viral VP1 proteins were detected *via* western blotting. β-actin served as a loading control. This result is representative of three independent experiments.

The mTOR/ULK1 signaling pathway plays a key role in mediating the initiation and formation of an autophagosome (26). The phosphorylation levels of mTOR were decreased in echovirus-infected cells (**Figure 5A**). Both levels of ULK1 and phosphorylated ULK1 protein on S757 were also decreased in virus-infected cells (**Figure 5A**). Upon autophagy induction, the ULK1 complex translocates to autophagy initiation sites and regulates the recruitment of a second kinase complex, the vacuolar protein sorting 34 (VPS34) complex consisting of VPS34, as well as Beclin-1, VPS15, and ATG14L, which promotes the formation of autophagic vesicles (26, 27). Therefore, we further checked the levels of VPS34 and Beclin-1. As shown in **Figure 5B**, both VPS34 and Beclin-1 protein levels increased in echovirus-infected cells in a dose-dependent manner. The results indicated that echovirus infection induced the activation of signaling pathways involved in autophagosome formation.

### Induction of autophagy promotes echovirus replication

**Figure 6:**
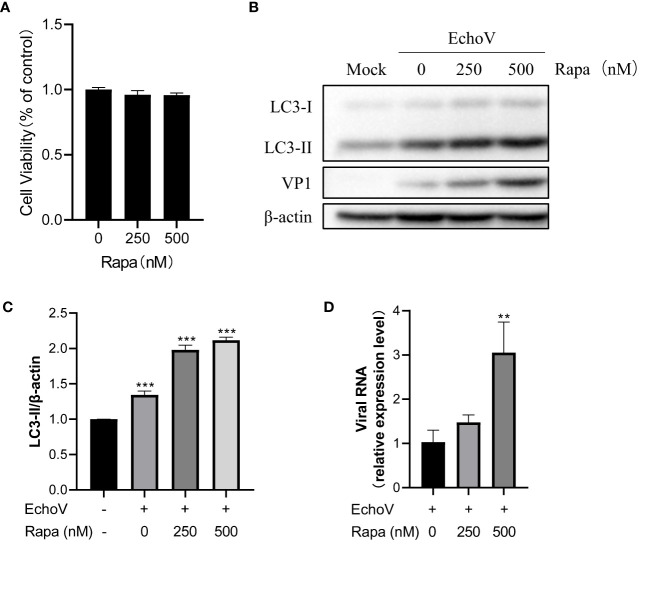
Induction of autophagy promotes Echovirus replication. RD cells were infected or not (Mock) with echovirus 11 at 0.1 MOI for 1.5 h and then untreated (Echo) or treated with rapamycin (Rapa) at indicated concentrations for 16 h. Cells were then harvested. **(A)** Cell counting kit-8 (CCK8) assay was performed to examine the cytotoxicity of rapamycin to RD cells. **(B)** Intracellular LC3 and viral VP1 proteins were detected *via* western blotting. β-actin served as a loading control. This result is representative of three independent experiments. The relative band intensities of detected proteins were calculated using ImageJ from NIH, and the result was represented as the ratio of LC3-II to β-actin **(C)**. **(D)** Total RNA was extracted from cells and subjected to a quantitative reverse transcription-polymerase chain reaction (qRT-PCR) to detect viral RNA. A one-way ANOVA was used to determine statistically significant differences in multiple comparisons. **P<0.01; ***P<0.001.

Previous studies have demonstrated that autophagy may serve an antiviral or proviral function during diverse viral infections (14). Therefore, to investigate the impact of autophagy induction on echovirus infection, we monitored virus expression and replication under autophagy inducer rapamycin treatment. Rapamycin at indicated concentrations showed no toxicity to RD cells (**Figure 6A**). Induction of autophagy through rapamycin treatment significantly increased viral protein VP1 expression in a dose-dependent manner (**Figures 6B–C**). Moreover, the levels of viral RNA were also increased under rapamycin treatment in a dose-dependent manner (**Figure 6D**). These results suggested that induction of autophagy promoted echovirus replication.

### Inhibition of autophagy impairs viral protein VP1 expression

**Figure 7:**
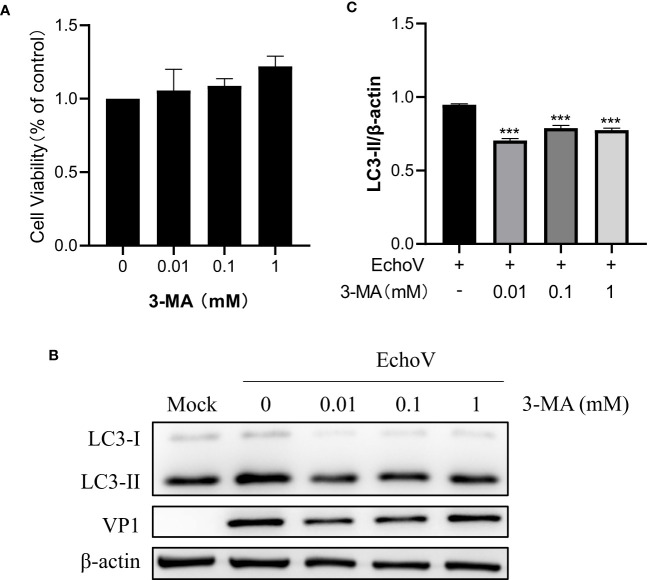
Inhibition of Autophagy inhibits EchoV replication. RD cells were pre-untreated or pre-treated with 3-MA at indicated concentrations for 2 h, followed by infected (EchoV) or not (Mock) with echovirus 11 at 0.1 MOI for 1.5 h. At 16 h post-infection, cells were then harvested. **(A)** Cell counting kit-8 (CCK8) assay was performed to examine the cytotoxicity of 3-MA to RD cells. **(B)** Intracellular LC3 and viral VP1 proteins were detected *via* western blotting. β-actin served as a loading control. This result is representative of three independent experiments. The relative band intensities of detected proteins were calculated using ImageJ from NIH, and the result was represented as the ratio of LC3-II to β-actin **(C)**. A one-way ANOVA was used to determine statistically significant differences in multiple comparisons. ***P<0.001.

To further validate the effect of autophagy on echovirus replication, 3-MA, a widely used selective autophagy inhibitor, was used. As shown in **Figure 7**, 3-MA at indicated concentrations showed no toxicity to RD cells (**Figure 7A**). Treatment with 3-MA impaired the level of LC3-II expression, suggesting the inhibition of autophagy (**Figure 7B**). Correspondingly, viral VP1 protein expression reduction was observed (**Figures 7C**). This finding confirmed that autophagy might serve a proviral function during echovirus infection.

## Discussion

**Figure 8:**
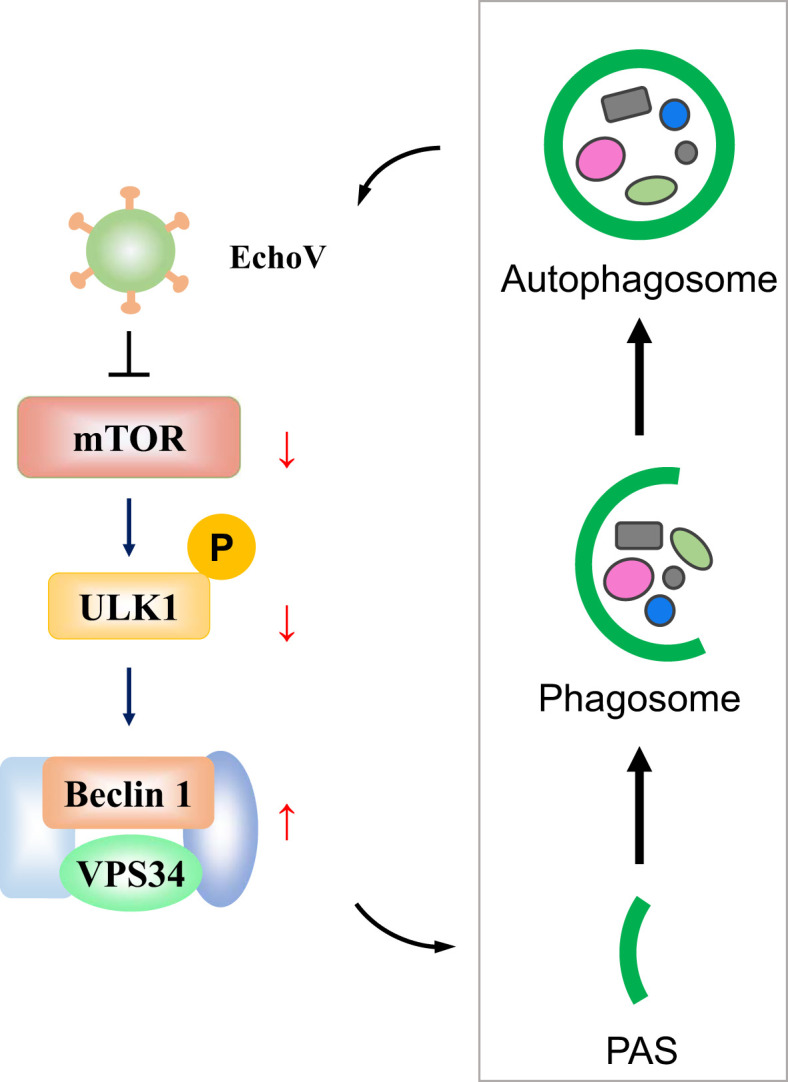
A proposed model depicting the interplay between echovirus infection and autophagy *via* regulating mTOR/ULK1 signaling pathway. Upon echovirus infection, the levels of phosphorylated mTOR and phosphorylated ULK1 protein on S757 decreased. Furthermore, both VPS34 and Beclin-1 protein levels increased, which promotes the formation of autophagic vesicles. Echovirus-induced autophagy promoted viral replication.

Autophagy is an evolutionarily conserved cellular process through which the lysosome could degrade long-lived proteins, damaged organelles, or invading pathogens to maintain cellular homeostasis and host health (13, 17). However, viruses have evolved strategies during a long evolutionary process by which they can hijack and subvert host autophagy to favor their benefits (14). Echovirus is one of the most common worldwide causes of severe illnesses in neonates or infants. The interplay between echovirus and autophagy needs to be better understood. Here, we demonstrated that echovirus 11 induced autophagy to promote its replication *via* regulating mTOR/ULK1 signaling pathway (**Figure 8**).

Previous studies have indicated that several core components of the autophagy machinery, including Beclin-1, Atg12, Atg14, Atg16L1, and LC3, are important for echovirus 7 entry into polarized Caco-2 cells (21). However, the impact of autophagy on echovirus replication after virus entry was not discussed in that paper. Here, our data provided experimental evidence that autophagy also participates in echovirus replication. Replication of positive-stranded RNA viruses requires intracellular membrane surfaces on which they assemble their replication complexes (28). Double-membrane compartments formed during autophagy can provide a physical platform for the viral replication machinery. For example, the influenza A virus triggers the accumulation of autophagosomes for viral replication (29). Zika virus infection results in membrane rearrangements and induction of autophagy, which are considered to be the sites of viral RNA replication and virion assembly (30). CVB3, another important enterovirus classified within the B species, also uses autophagy for replication (31). Similar to these results, echovirus infection can also induce autophagy. In virus-infected RD cells, autophagosome can be observed. Presumably, echovirus exploited the autophagic membrane to support its replication.

Mechanically, membrane compartments formed during autophagy can locally concentrate essential intermediates and protect viral RNAs from detection by innate immune sensors and degradation (14). For example, HCV induces autophagosome formation but blocks lysosomal fusion, resulting in the accumulation of autophagosomes in support of HCV replication (32). CVB3-induced accumulation of autophagosomes *via* blockage of autophagosome-lysosome fusion (33). However, in this study, p62, a marker of autophagy-mediated protein degradation or autophagic flux, decreased during echovirus infection, indicating that echovirus infection may not interfere with the fusion of autophagosomes with lysosomes. Therefore, further studies may be needed to elucidate the mechanism by which echovirus-induced autophagy promotes viral replication. Notably, the expression level of viral VP1 protein increased upon autophagy induction and decreased upon autophagy inhibition. This result suggested that autophagy may play a role in viral protein synthesis.

The process of autophagosome formation is tightly controlled. The serine/threonine protein kinase mTOR is one of the key regulators and negatively controls autophagosome formation (34). We consistently showed that mTOR phosphorylation levels decreased after echovirus infection, suggesting that echovirus-induced autophagy is triggered by mTOR dephosphorylation. Furthermore, we observed that the level of phosphorylated ULK1 on S757 decreased. As phosphorylation of the major autophagy activator ULK1 on S757 by mTORC1 inhibits ULK1 activity and represses autophagy (35), our findings thus suggested that mTOR/ULK1 axis participates in echovirus-induced autophagy. Subsequently, Beclin-1 (a homolog of yeast ATG6) is the first identified mammalian autophagy protein critical for the signaling pathways involved in autophagosome formation (27). Some viral proteins, like hepatitis B virus X protein, can sensitize cells to starvation-induced autophagy *via* up-regulation of Beclin-1 expression. In the present study, we also observed the up-regulation of Beclin-1 during echovirus infection. In addition, Beclin-1 forms the class III PI3K complex with VPS34, which promotes autophagosome formation (36). Our data also showed that the levels of VPS34 increased upon echovirus infection. These results further demonstrated that echovirus could induce autophagy *via* activating autophagy signaling. Our preliminary data showed that VP1, a capsid protein of echovirus, did not participate in echovirus-induced autophagy. Therefore, further studies may be needed to elucidate the mechanism by which echovirus infection regulates mTOR/ULK1 signaling pathway.

In conclusion, our study demonstrates that autophagy can be induced by echovirus infection and exhibits a proviral function, revealing the potential role of autophagy in echovirus infection. These findings not only shed light on the molecular mechanisms underlying how echovirus hijacks cellular components and pathways for its benefits but also provide therapeutic options against echovirus infection.

## Data availability statement

The original contributions presented in the study are included in the article/supplementary materials. Further inquiries can be directed to the corresponding author.

## Author contributions

CW and JX designed the research, wrote and revised the paper. LZ, WY, YM and YL performed the experiments. QW, SL, GP and ZZ analyzed the data. All authors contributed to the article and approved the submitted version.
